# Early radical cystectomy with negative margins in a 57-year-old female with myxoid/round cell liposarcoma of the bladder suggests prolonged overall survival

**DOI:** 10.1186/s12301-021-00152-y

**Published:** 2021-03-23

**Authors:** Johannes Eduard Delport, Khanyisa Makamba

**Affiliations:** 1 Medical Officer, Department of Urology, Port Elizabeth Provincial Hospital, Buckingham Road, Mount Croix, Port Elizabeth, Eastern Cape South Africa; 2 Head of Clinical Unit, Department of Urology, Port Elizabeth Provincial Hospital, Buckingham Road, Mount Croix, Port Elizabeth, Eastern Cape South Africa

**Keywords:** Liposarcoma, Bladder cancer, Oncology

## Abstract

**Background:**

Bladder cancer is the 17th most common cancer in the female population. Most bladder cancers are of urothelial origin. Sarcomas of the bladder are very uncommon.

**Case presentation:**

This case concerns a 57-year-old female from a rural town in the Eastern Cape who was diagnosed with myxoid/round cell liposarcoma of the bladder. After the initial transurethral resection of the bladder tumour, she underwent an anterior exenteration with Bricker’s diversion (ileal conduit) and negative margins were achieved.

**Conclusions:**

Sarcomas of the bladder are known to have a poor prognosis. Our patient is alive with good stoma function more than 24 months since her radical surgery. We are of the opinion that her favourable overall survival is attributable to early radical surgical intervention with negative margins.

## Background

Of all human cancers, bladder cancer ranks 17th among the commonest in the female population with an incidence of approximately 70,000 cases per year [1]. The most common histological subtypes of bladder cancer include urothelial carcinoma (± 90%), squamous cell carcinoma (± 5%), and adeno carcinoma (± 2–3%). Some of the rarer cancers, however, also include sarcomas. This case report focuses on the latter and discusses the treatment of a 57-year-old female with myxoid/round cell liposarcoma of the bladder.

## Case presentation

In July 2018, a female patient (who resided in a small village in rural Eastern Cape) was referred to the outpatients department of the Port Elizabeth Provincial Hospital. The referring doctor was concerned about her persistent microhematuria and dysuria. On having her history taken, the patient mentioned a long period (± 30 years) of intermittent macrohematuria, along with occasionally passed clots. This was associated with pain over the bladder and with lower urinary tract symptoms (LUTS) in the form of urgency, urgency-incontinence, and dysuria. She reported no history of smoking, and she had been a housewife all her life with no obvious occupational exposure to carcinogens. She had well-controlled hypertension and no other known comorbidities. Her chronic medication included daily calcium-channel blockers, thiazide diuretics, and aspirin. Regarding gynaecological history, her menopause started in her mid-40 s, she has 6 children (all normal vaginal deliveries without complications) and had a bilateral tubal ligation in 1991. She reported no other surgical history as well as no family history of cancer.

On clinical examination, she appeared healthy with stable vitals and a soft abdomen with adiposity and no palpable masses or organomegaly. Her vaginal examination revealed no abnormalities with no notable masses, blood, or observed incontinence. Her urine dipstick demonstrated microhematuria. A urine specimen was subsequently sent to our laboratory for microscopy, culture, and sensitivity (MCS) testing. The laboratory findings included numerous leukocytes and erythrocytes, a bacterial urinary tract infection (UTI), namely Escherichia coli and Enterococcus faecalis, but no Schistosoma ova.

**Fig. 1:**
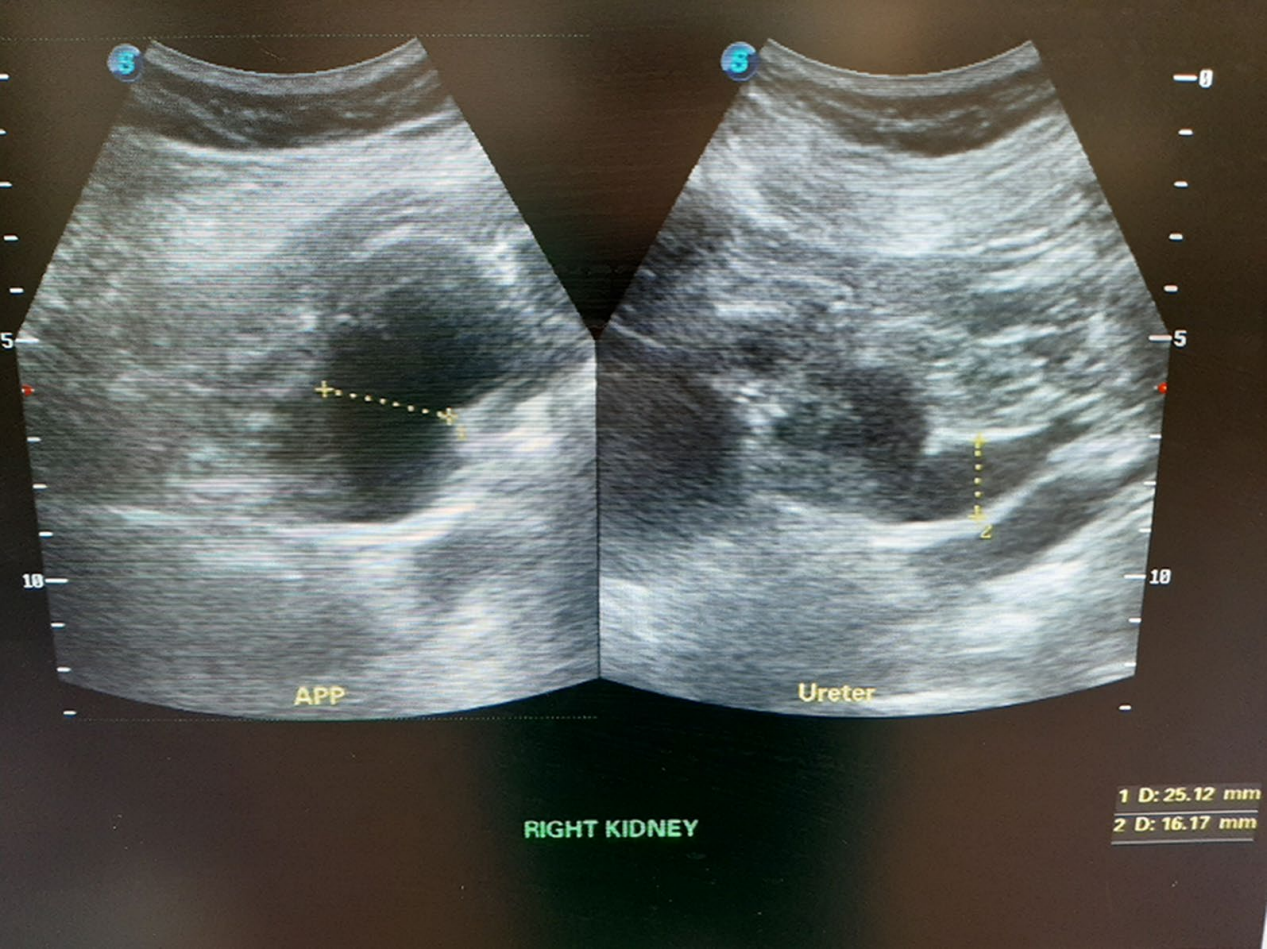
Ultrasound with right hydroureteronephrosis

**Fig. 2:**
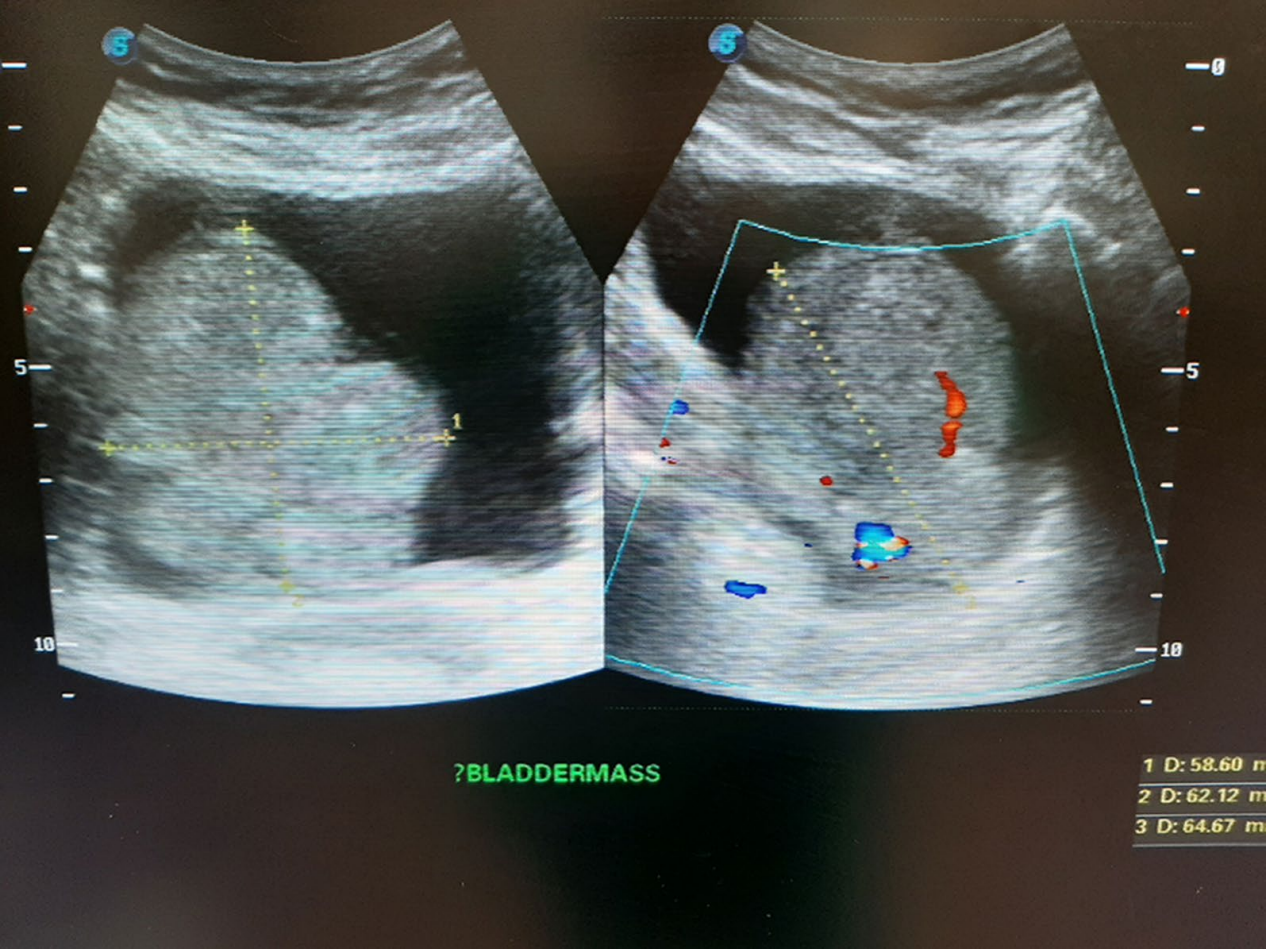
Ultrasound with bladder mass

A kidney-ureter-bladder (KUB) ultrasound revealed right hydroureteronephrosis and a large, irregularly shaped, heterogenous mass with internal vascularity at the base of the bladder (Figs. 1 and 2).

**Fig. 3:**
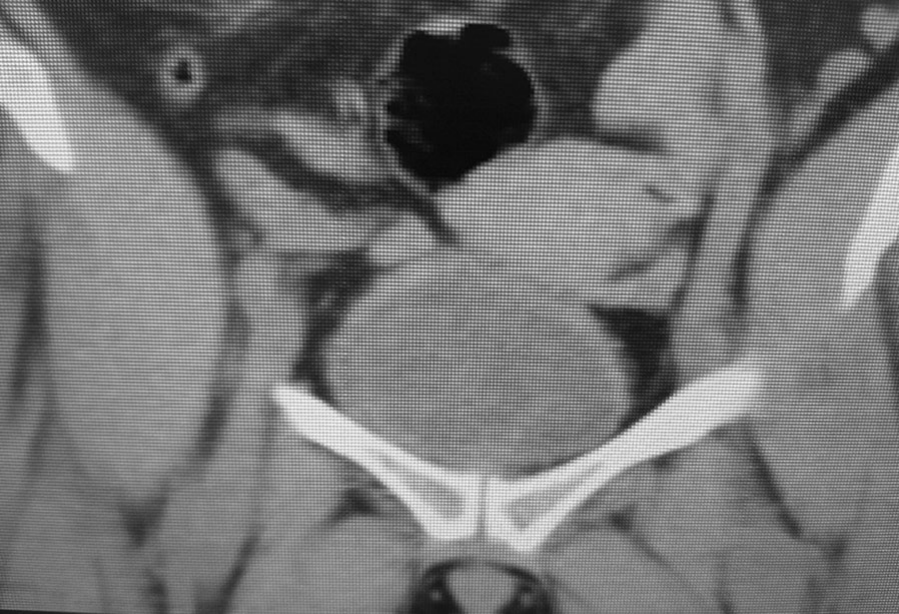
Noncontrasted CT with bladder mass

**Fig. 4:**
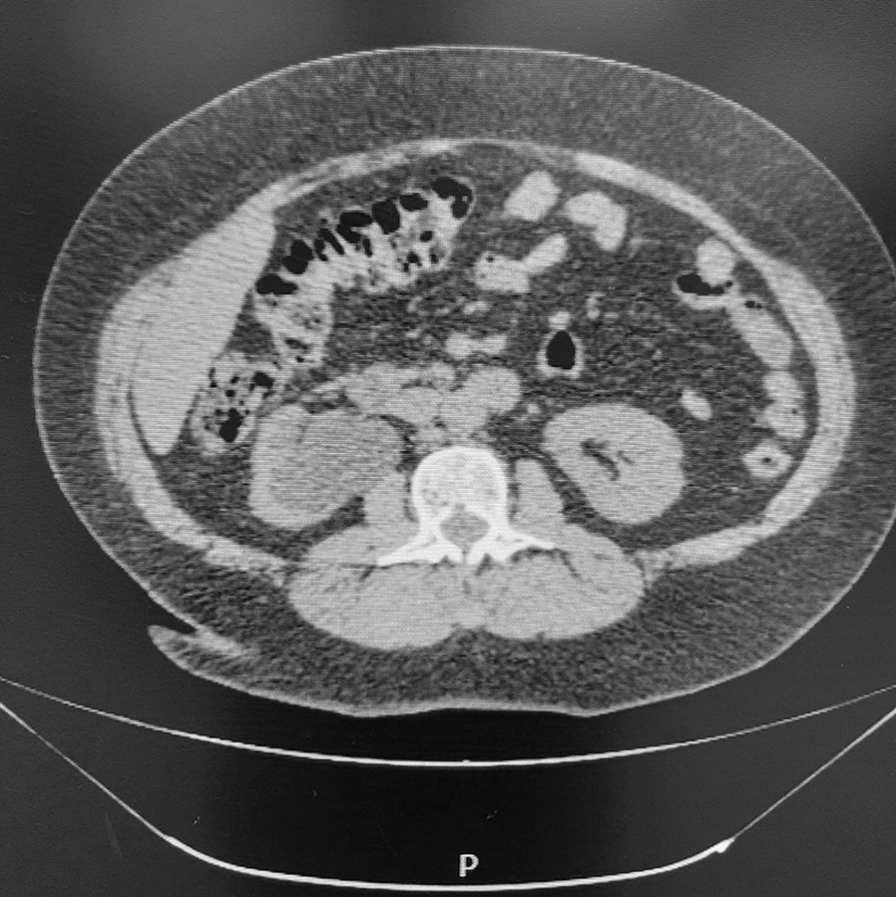
Noncontrasted CT with right hydroureteronephrosis

Her renal function was deranged (Creatinine = 104 micromol/L; estimated glomerular filtration rate = 47 ml/min/1.73 m^2^), most likely because of her chronic hypertension, and this complicated matters regarding further imaging. The ideal imaging of choice would have been a contrasted computed tomography (CT) of her abdomen and pelvis, but we did not want to risk further renal deterioration because of contrast nephropathy. At our resource-challenged facility, we had a very long waiting period for magnetic resonance imaging (MRI) and therefore proceeded with a noncontrasted computed tomography scan of the abdomen and pelvis in order to prevent further delays in definitive treatment. The results confirmed the presence of the right hydroureteronephrosis and a bladder mass of 6.3 cm by 6.9 cm (Figs. 3 and 4). No metastatic spread was noted, and her chest X-ray was clear.

During the initial white light cystoscopy, multiple blood clots were evacuated, and a large smooth-walled tumour of unidentifiable origin was noted. An incomplete TURBT was done because of the high tumour burden along with impaired visibility experienced during resection.

The histology report of the TURBT noted that sections showed a malignant tumour composed of cellular areas which, in turn, consisted of round cells. The round cell component constituted more than 40% of the tumour in these sections. Additional myxoid areas also contained ovoid cells, of which some indicated convincing adipocytic differentiation. A prominent branching capillary pattern was seen in these areas. Lipoblasts were also noted. The bladder tumour was classified as Fédération Nationale des Centres de Lutte le Cancer (FNCLCC) grade 2.

With regard to immunohistochemistry, the tumour stained positive for Vimentin, S100 and CD10; and stained negative for 34BE12, CK7, CK20, p63, CK5/6, CD45, CD117, GATA3, Desmin, SMA, P504S, Melan A and HMB45. The Ki-67 proliferation index was ± 10–15%.

A diagnosis of myxoid/round cell liposarcoma of the bladder was made. Subsequently, it was decided to perform an anterior exenteration and Bricker’s diversion (ileal conduit). Although an anterior exenteration is regarded as having multiple complications (intra- and post-operatively), the surgery was successful with no adverse intra-operative events. The histology of the specimen confirmed the initial diagnosis of myxoid/round cell liposarcoma of the bladder. Infiltration into the muscularis propria with numerous lipoblasts in a myxoid stroma was also noted. The tumour did not extend through the muscularis propria, and there was no vascular or perineural invasion. The adnexa, distal ureters, pelvic lymph nodes, and uterus were all tumour-free. There was an incidental finding of a leiomyoma of the uterus.

Post-operatively, the patient’s stay was unfortunately complicated by chronic diarrhoea with profound hypokalaemia. Her condition was further complicated by an acute kidney injury (AKI). She also developed venous thrombo-embolism (despite prophylaxis with subcutaneous unfractionated heparin), with bilateral pulmonary emboli and ileo-femoral thrombi. After a couple of weeks, the patient recovered from the diarrhoea and AKI. She received anti-coagulation therapy and had an inferior vena cava filter inserted by the hospital’s vascular surgeons. The patient was also diagnosed with HIV and an Absolute CD4 count of 333. She was thus referred to the Internal Medicine department of the same hospital for initiation of anti-retroviral therapy.

During further long-term follow up consultations at our facility, the patient developed a single UTI for which she was treated. There have been no stoma-related complications, and, at present, the stoma is functioning well. No local or regional recurrence of the tumour has been noted with follow up CT scans. More than 24 months since radical surgery, it can be confirmed that the patient is alive and well.

## Discussion

Sarcomas of the bladder are very rare, even more so a liposarcoma of the bladder. This is emphasised by a review of 1583 adult soft tissue sarcomas, of which only 10 had tumours that originated in the bladder [2]. Furthermore, according to a review of 590 cases of visceral sarcomas, only 13 liposarcomas were found to have been diagnosed and none of them were myxoid/round cell liposarcoma [3].

The etiology of sarcomas is mostly idiopathic with ionizing radiation having been identified as the most common causative environmental factor. Some genetic syndromes associated with sarcomas include retinoblastoma, neurofibromatosis type 1, familial adenomatous polyposis syndrome, and Li-Fraumeni syndrome [4]. Bladder leiomyosarcoma following cyclophosphamide therapy has, however, also been ported with the likely causative agent having been identified as acrolein, a cytotoxic metabolite of cyclophosphamide, excreted in urine [5]. This possible complication may need to be considered in patients who receive cyclophosphamide therapy. It is unknown whether any cases of bladder liposarcoma developing secondary to chemotherapy have been described yet.

According to Levy et al., radiologically, myxoid liposarcoma contains an abundant myxoid matrix with a high water content. On CT imaging, it appears hypoattenuating. Magnetic resonance imaging (MRI) findings include hypointense lesions on T1-weighted images and hyperintense lesions on T2-weighted images. Enhancement of the myxoid matrix is an important feature that distinguishes this from a cystic mass and is better demonstrated with MRI rather than with a CT scan. Characteristically, thick enhancing septa, which correspond to fibrous bands running through the myxoid stroma, course through the mass. An enhancing patchy or nodular soft tissue component that demonstrates intermediate signal intensity on T1- and T2-weighted MRI images is also present. Areas of mature fat may be identified within the lesion [6]. About 30% of liposarcomas are myxoid/round cell liposarcomas which represent a single entity with a variable round cell component [7].

The foundation for definitive treatment of operable bladder liposarcoma is surgical resection that ensures negative surgical margins. In the case of metastatic disease, however, soft tissue sarcomas are treated mostly with palliative cytotoxic chemotherapy. In such cases, the chemotherapy agent of choice for myxoid liposarcoma is doxorubicin-based chemotherapy [8].

Three important prognostic factors for adult genito-urinary sarcomas have been identified: site; size; and grade of tumour [9]. According to Kilpatrick et al*.*, the aggressiveness of myxoid liposarcomas is determined by the contribution of the round cell component, where more than 5% of such round cell component carries a poor prognosis [10]. A typical case of such primary round cell bladder liposarcoma in an 80-year-old female has been reported. The patient passed away 10 months after the diagnosis was made [11].

From the above discussion, it is clear that the prevalence of myxoid/round cell liposarcoma is sporadic and the prognosis is weak. Once accurately diagnosed, doctors may thus be confronted with difficult decisions in terms of management and treatment.

## Conclusion

In general, the prognosis of any bladder sarcoma is poor. Sufficient convincing data regarding bladder sarcoma response to chemotherapy and/or radiotherapy are scarce, given the paucity of cases. More specifically, effective management and treatment of myxoid/round cell liposarcoma, as a rare and uncommon tumour of the bladder, is unclear. Our case report suggests that early surgical control with negative surgical margins could be considered a potential intervention to improve patient overall survival.

## Data Availability

None.

## Abbreviations

LUTS: Lower urinary tract symptoms
MCS: Microscopy, culture, and sensitivity
UTI: Urinary tract infection
TURBT: Transurethral resection of bladder tumour
FNCLCC: Fédération Nationale des Centres de Lutte le Cancer
AKI: Acute kidney injury
HIV: Human immunodeficiency virus
MRI: Magnetic resonance imaging
